# Correlation and Agreement Between the SCORE2 and PREVENT 10-Year Atherosclerotic Cardiovascular Disease Risk Scores: Insights from Coronary Computed Tomography Angiography

**DOI:** 10.3390/diagnostics14232625

**Published:** 2024-11-22

**Authors:** Mehmet Emre Ozpelit, Ayse Colak, Hatice Irem Uzumcu, Zeynep Kumral, Ebru Ozpelit

**Affiliations:** 1 Department of Cardiology, Izmir Economy University, Izmir 35330, Türkiye; 2 Department of Cardiology, Dokuz Eylul University, Izmir 35330, Türkiye; ayse.colak@deu.edu.tr (A.C.); ebru.ozpelit@deu.edu.tr (E.O.); 3 Private Ahu Hospital, Mugla 48700, Türkiye; irem.uzumcu@gmail.com; 4 Unye State Hospital, Ordu 52300, Türkiye; zeynepkumral@gmail.com

**Keywords:** Systemic Coronary Risk Estimation 2, Predicting Risk of CVD EVENTs, coronary computed tomography angiography, coronary artery calcium score

## Abstract

**Background**: We aimed to evaluate the correlation and agreement between the Systemic Coronary Risk Estimation 2 (SCORE2) and Predicting Risk of CVD EVENTs (PREVENT) 10-year ASCVD risk scores by incorporating computed tomographic (CT) data to assess differences between the scoring systems. **Methods**: The PREVENT risk score was calculated for 171 patients, while the SCORE2 and SCORE2 Older Persons (OP) risk scores were calculated for 113 patients. Coronary artery calcium (CAC) scores were calculated, and the grading of coronary artery disease (CAD) was assessed according to these scores. **Results**: According to the PREVENT risk category, 79 patients (46.2%) were in the low-risk category, 32 (18.7%) were in the borderline-risk category, and 51 (29.8%) were in the intermediate-risk category. In contrast, the SCORE2 systems placed 32 patients (28.3%) in the low- to moderate-risk categories. Only 9 patients (5.3%) were classified as being at high risk by PREVENT, while SCORE2 categorized 39 patients (34.5%) as being at high risk and 42 patients (37.2%) as being at very high risk. There was a strong correlation between the scores (r = 85, *p* < 0.001), with a Bland–Altman plot analysis showing a bias of −3.71 points and the limits of agreement ranging from −16.06 to 8.64. The total CAC score and CAD grading were significantly different across the PREVENT risk groups (*p* < 0.001 for all) but were similar across the SCORE2 groups (*p* = 0.3 and *p* = 0.051, respectively). **Conclusions**: There is a strong correlation and agreement between the two risk scores. However, SCORE2 tends to categorize more patients as high-risk than PREVENT does. Additionally, the PREVENT risk categories are more effective than SCORE2 in determining the likelihood of CAD based on CT results.

## 1. Introduction

Cardiovascular diseases (CVDs) are still leading causes of morbidity and mortality, and predicting CVD risk is a cornerstone of CVD prevention [1]. Recent guidelines on CVD prevention from the European Society of Cardiology (ESC) recommended the usage of Systemic Coronary Risk Estimation 2 (SCORE2) for healthy individuals aged 40–69 years and SCORE2-Older Persons (SCORE2-OP) for apparently healthy individuals aged ≥70 years for determining the 10-year risk of fatal and nonfatal CVD (myocardial infarction (MI), stroke) [1]. Similarly, the current American Heart Association (AHA) and American College of Cardiology (ACC) guidelines on primary prevention recommended pooled cohort equations (PCEs) for predicting 10-year atherosclerotic CVD (ASCVD) (nonfatal MI, nonfatal stroke, and fatal CVD) [2].

Both SCORE2 systems are currently used in our country, and they incorporate gender, age, active smoking, systolic blood pressure (BP), and non-high-density lipoprotein (HDL) cholesterol data for risk calculation. However, several conditions, such as chronic kidney disease (CKD) and obesity, often increase the likelihood of ASCVD [1]. It has been shown that kidney function decreases nearly 10 mL/min/1.73 m^2^ per decade even in the absence of CKD, and the probability of ASCVD rises significantly below an estimated glomerular filtration (eGFR) of approximately 75 mL/min/1.73 m^2^ [3]. Similarly, a higher body mass index (BMI) was found to be significantly related to ASCVD in both sexes [4].

Recently, cardiovascular–kidney–metabolic (CKM) syndrome has been outlined by the AHA, and this syndrome is related to an increased load of CVD, including ASCVD and heart failure (HF) [5]. As deterioration in CKM health is linked with earlier initiation of CVD and there are emerging treatment options that protect both cardiovascular and kidney health [6], newer risk prediction tools that incorporate CKM parameters such as eGFR and BMI in scoring systems are warranted to decrease total CVD risk via these novel therapies.

For this purpose, the Predicting Risk of CVD EVENTs (PREVENT) scoring system was developed in the United States; it predicts the 10- and 30-year risk of total CVD (composite of fatal and nonfatal ASCVD and HF), the 10- and 30-year risk of ASCVD (MI, fatal coronary artery disease (CAD), and stroke), and the 10- and 30-year risk of HF [7].

This work aimed to examine the correlation and agreement between the SCORE2 and newer PREVENT 10-year ASCVD risk scores in a known ASCVD-free cohort by incorporating coronary computed tomography angiography (CCTA) data to assess whether there were differences between the groups and the scoring systems in terms of CCTA parameters.

## 2. Materials and Methods

### 2.1. Patients

We retrospectively enrolled 171 consecutive individuals who presented at an outpatient clinic with chest pain and underwent CCTA because they were considered to have a low clinical likelihood of CAD by the clinician between January 2018 and December 2021. Patients aged between 30 and 79 years were included to calculate the PREVENT risk score. Patients with a high likelihood of CAD, current usage of lipid-lowering agents, familial hypercholesterolemia, known ASCVD, left ventricular ejection fraction of <50%, and eGFR < 45 mL/min/1.73 m^2^ were excluded from the analysis.

The baseline data included demographics, anthropometric measurements, systolic and diastolic BP, and laboratory data, including the lipid profile. Hypertension (HT) was characterized by a systolic BP of 140 mmHg or higher, a diastolic BP of 90 mmHg or higher, or the current use of antihypertensive medication. Diabetes mellitus (DM) was identified using a fasting serum glucose level of 126 mg/dL or greater or the use of medication for DM management. A family history of ASCVD was outlined as having first-degree male relatives who developed the condition before age 55 or first-degree female relatives who developed it before age 65.

### 2.2. Risk Score Calculation

The PREVENT risk score was determined using the PREVENT^TM^ online calculator [8]. Patients were categorized according to their scores as low-risk (<5%), borderline risk (5% to 7.4%), intermediate-risk (7.5% to 19.9%), and high-risk (≥20%). The PREVENT risk score was calculated for all 171 patients.

The SCORE2 and SCORE2-OP risk scores (SCORE2 systems) were calculated following the recent ESC guidelines on CVD prevention (1). High-risk charts were utilized because Turkey is a high-risk region according to the World Health Organization’s cardiovascular mortality rates. The SCORE2 risk score was used for patients aged 40–69 years, and the SCORE2-OP risk score was used for patients aged 70–79 years without known ASCVD and DM. The SCORE2 system risk score was calculated for 113 patients because of the limit of age and the presence of DM. Patients were divided into 3 categories—low- to moderate-risk, high-risk, and very-high-risk—according to their scores and age [1].

### 2.3. The Assessment of CCTA Data

The coronary atherosclerotic burden was assessed with the coronary artery calcium (CAC) score using multidetector (640-slice) CT scanners (Toshiba, Aquilion ONE, Tokyo, Japan). Beta-blockers were used when appropriate (heart rate > 70 bpm/min). Images were acquired after a bolus injection of 57 mL of contrast agent (Iohexol) with ECG triggering. The scan slice thickness was 0.5 mm. A skilled cardiologist and a radiologist (dual reporting) analyzed all scans after processing on a workstation (Vitrea, Dania Beach, FL, USA), and high-grade coronary stenosis was defined as ≥70%. CAC scores were expressed as absolute values and graded according to these values as no evidence of CAD (0), minimal (0–9), mild (10–99), moderate (100–399), severe (400–999), and extensive (≥1000) [9].

### 2.4. Data Analysis

SPSS version 27 (SPSS, Inc., Chicago, IL, USA) was utilized for data analysis. The normality of continuous variables was assessed with the Kolmogorov–Smirnov test and reported as means ± standard deviations or medians with interquartile ranges. Categorical variables were presented as counts and percentages. Nonparametric tests were applied for variables with skewed distributions. To compare the PREVENT and SCORE2 categories, the Kruskal–Wallis test was used for continuous data, and the Chi-square test was applied to categorical data. The relationship and agreement between the PREVENT and SCORE2 systems were analyzed using Pearson’s correlation coefficient and Bland–Altman plots, with 95% limits of agreement set as ±1.96 standard deviations from the mean difference. A post hoc power analysis was conducted using G Power version 3.1.9.7 to compare total CAC scores between the PREVENT and SCORE2 risk category groups. A *p*-value less than 0.05 was deemed statistically significant.

## 3. Results

### 3.1. Clinical and Laboratory Findings

The baseline demographic and laboratory data of all 171 subjects are presented in Table 1. The median age of the whole cohort was 53.7 ± 13.3 years, and 52 (30.4%) of the patients were female. HT was present in 39.2% (*n* = 67), and DM was present in 21.6% (*n* = 37) of the patients. The mean total cholesterol level was 231.7 ± 52.9 mg/dL, and the mean low-density lipoprotein (LDL) cholesterol level was 140.7 ± 43.4 mg/dL. The median triglyceride level was 150 (98–255) mg/dL, and the median HDL cholesterol level was 48 (39–59) mg/dL.

### 3.2. CCTA Findings and Risk Category Data

The CCTA findings and the risk categories according to the PREVENT and SCORE2 systems are presented in Table 2. The PREVENT risk scores were available in all patients (*n* = 171), whereas the SCORE2 scores were available in 113 of the patients. Half of the patients (50.6%) had no evidence of CAD according to the total CAC score. Seventy-nine (46.2%) of the patients were at low risk, thirty-two (18.7%) of the patients were at borderline risk, and fifty-one (29.8%) of the patients were at intermediate risk according to the PREVENT 10-year CVD risk category. However, 32 (28.3%) of the patients were in the low- to moderate-risk category according to the SCORE2 and SCORE2-OP 10-year CVD risk categories. Only nine (5.3%) of the patients were at high risk according to the PREVENT 10-year CVD risk category, whereas the SCORE2 system placed thirty-nine (34.5%) of the patients in the high-risk category and forty-two (37.2%) of the patients in the very-high-risk category.

### 3.3. Correlation and Degree of Agreement Between the Two Risk Scores

**Figure 1:**
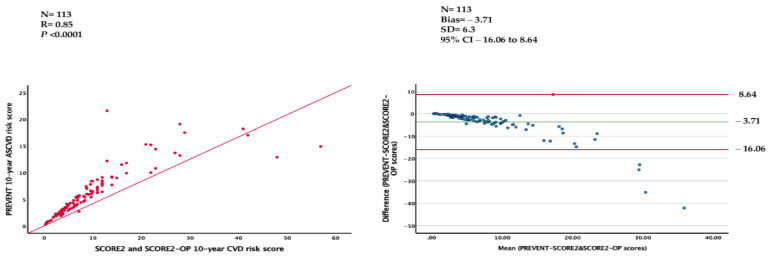
Correlation and Bland–Altmann analyses between PREVENT 10-year ASCVD and SCORE2-SCORE2-OP 10-year CVD risk scores.

Correlation and Bland–Altman analyses were performed in 113 patients. The PREVENT 10-year ASCVD risk scores were strongly correlated with SCORE2 system 10-year CVD risk scores (r = 0.85, *p* < 0.001), and the Bland–Altman plot analysis showed a bias of −3.71 points. The limits of agreement were −16.06 and 8.64 with the 95% CI (Figure 1). The correlation and agreement between the two scoring systems were good, but the risk categories were significantly different (*p* < 0.0001) (Table 3).

Among the 113 patients in whom both score risk categories were available, 58 (51.3%) of the patients were at low risk, 22 (20.4%) of the patients were at borderline risk, 31 (27.4%) of the patients were at intermediate risk, and 1 (0.9%) of the patients was in the high-risk category according to the PREVENT risk score (Table 3). All 32 of the low- to moderate-risk patients according to the SCORE2 system risk categories were in the low-risk category according to the PREVENT risk score. However, none of the 39 high-risk patients according to the SCORE2 system risk categories were in the high-risk category according to the PREVENT risk score. Additionally, the majority of the very-high-risk patients according to the SCORE2 system risk categories were in the intermediate-risk category (*n* = 29, 93.5%) according to the PREVENT risk score.

### 3.4. Differences Between CCTA Data Across PREVENT and SCORE2/SCORE2-OP Risk Categories

**Figure 2:**
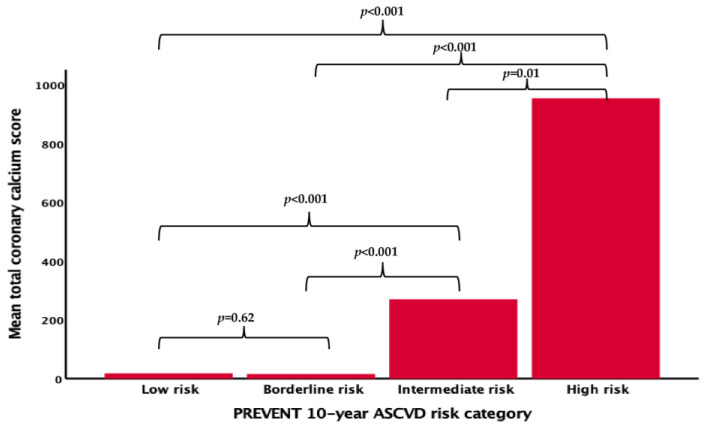
Computed tomographic findings according to PREVENT 10-year ASCVD risk categories.

The differences between CCTA findings according to the PREVENT risk score categories were available in 171 patients and are presented in Table 4 and Figure 2. The effect size for comparing total CAC scores in the PREVENT risk groups was calculated as 0.376 and power analysis revealed a power of 0.99 for the PREVENT risk score. The total CAC score and the grade of CAD according to this score were significantly different across the groups (*p* < 0.001 for all). The total CAC score was significantly different among all categories except for the low- and borderline-risk (*p* = 0.62) groups. The majority of low- and borderline-risk patients had no evidence of CAD (68.4% and 62.5%, respectively), and 44% of the high-risk patients had extensive CAD. High-grade coronary stenosis was significantly higher in the high-risk category (*p* < 0.001).

**Figure 3:**
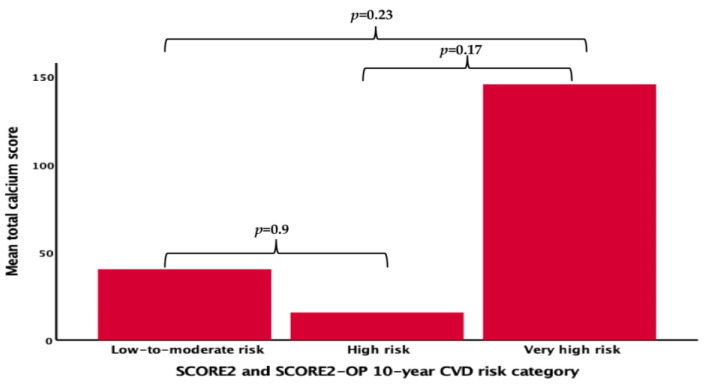
Computed tomography findings according to SCORE2 and SCORE2-OP 10-year CVD risk categories.

The differences in the CCTA findings according to SCORE2 system risk score categories were available in 113 patients and are presented in Table 5 and Figure 3. The effect size for comparing total CAC scores in the SCORE2 risk groups was calculated as 0.228 and power analysis revealed a power of 0.56 for SCORE2. The total CAC score and grading of CAD according to this score were similar across the groups (*p* = 0.3 and *p* = 0.051, respectively). The majority of low- to moderate-risk patients (62.5%) had no evidence of CAD, and approximately one-third of the very-high-risk patients had mild CAD (28.6%) according to the total coronary calcium score grade. High-grade coronary stenosis was similar across all risk categories (*p* = 0.37).

## 4. Discussion

The result of this study has two main foci; (1) the correlation of SCORE2 and PREVENT risk scores with each other and (2) the correlation of SCORE2 and PREVENT risk scoring systems with CCTA findings separately.

In respect of our first focus, the results of this study showed that there is a good correlation and agreement between PREVENT 10-year ASCVD and SCORE2/SCORE2-OP 10-year CVD absolute risk scores in patients without known ASVCD and DM. However, the risk categories were significantly different between the two scoring systems. All the low- to moderate-risk category patients according to the SCORE2 system were in the low-risk category according to the PREVENT risk system. However, most of the very-high-risk patients according to the SCORE2 system were in the intermediate-risk category according to the PREVENT risk system. These results indicate a disagreement between the scoring systems in respect of the categorization of the patients. The SCORE2 system appears to categorize patients as having a higher risk compared to the PREVENT risk scoring system.

For the second focus, the PREVENT risk categorization showed a good correlation with CCTA findings in respect of the total CAC score and the prevalence of high-grade stenosis. Most patients in the low- and borderline-risk categories according to PREVENT scoring system had no evidence of CAD according to CAC scores, whereas almost half of the high-risk patients had extensive CAD. The prevalence of high-grade coronary stenosis was also significantly higher in the high-risk category in the PREVENT risk system. However, the small number of high-risk patients in our analysis limits the ability to generalize these findings. In contrast, no significant correlation was found between the SCORE2 risk categories and CCTA findings. CAC scores and the prevalence of high-grade stenosis did not differ between the different risk categories of the SCORE2 system.

We showed that adding variables such as BMI, eGFR, and the use of antihypertensive drugs, which are not in the SCORE2 risk system but are in the PREVENT risk system, did not significantly change the individual scores of patients. It was previously demonstrated that the addition of BMI to risk scores did not significantly improve risk prediction [10]. We also showed that adding eGFR did not significantly alter the absolute risk score value. The main difference between the two risk scores arose from the change in risk category. Although we do not have a clinical event end point in our analyses, CCTA findings may be evaluated as a surrogate end point, indicating a better agreement of the PREVENT risk categorization with clinical data than the SCORE2 risk categorization.

One of the main challenges of risk prediction tools is model validity in real life in different populations. The development and external validation of the SCORE2 systems mostly originated in European regions [11]; therefore, the SCORE2 risk system can cause an over- and/or underestimation in other countries [12,13,14]. It has been demonstrated that the performance of the SCORE2 risk model depended on age groups in a Canadian population and the SCORE2-OP risk system overestimated the risk in this population [12]. Additionally, both SCORE2 and SCORE2-OP risk models overestimated the risk in an East Asian population [13]. Moreover, in Russia, a very-high-risk area, the SCORE2 risk system has been shown to accurately estimate risk in men but overestimate it in women [14]. There is no validation study for the SCORE2 risk system for Turkey. Based on our results, it is impossible to conclude that the SCORE2 risk system overestimates the risk in our country as we lack clinical endpoint data. However, it can be concluded that the higher risk designation in SCORE2 system does not align with the likelihood of CAD, as indicated by CCTA results. ’Since the PREVENT risk system is a newly developed model, there are no validation studies in the literature outside of the United States yet. These data are among the first in the literature to compare the SCORE2 and PREVENT risk assessment systems, as well as to provide a partial validation of the PREVENT score in assessing the likelihood of CAD.

As the defined risk categories in prediction risk models do not always allow for a clear conclusion on the necessity for starting lipid-lowering therapy, potential imaging risk modifiers such as CAC score and carotid ultrasound were recommended in recent ESC CVD prevention guidelines to reclassify patients [1]. It has been demonstrated that the inclusion of the CAC score in classic risk assessment tools has the most powerful impact on proper reclassification [15]. Based on our results, which demonstrated a high prevalence of zero calcium on CCTA in the high- and very-high-risk group according to the SCORE2 risk system, it is reasonable to use CCTA as a risk modifier before initiating treatment in Turkey. Overestimating the risk might lead to higher treatment costs and unnecessary interventions, and underestimating the risk may result in overlooking high-risk patients, leading to insufficient preventive strategies for these patients.

Our study has several potential limitations. The main limitations are its retrospective design and the small sample size. While the power for comparing total CAC scores across PREVENT risk groups was high (>0.80), the power for comparing CAC scores across SCORE2 risk groups was low (<0.80). As a result, the robustness of the findings for SCORE2 risk groups remains inconclusive. Additionally, the number of high-risk patients in the PREVENT risk scoring system was notably low, which was unexpected, especially since the majority of high-risk patients in the SCORE2 system were classified as intermediate risk in the PREVENT system. A larger sample size would be needed to strengthen these findings. Another key limitation is the lack of data on clinical events. The validation of each scoring system in our population should be carried out with larger cohorts and prospective data.

## 5. Conclusions

There is good correlation and agreement between the PREVENT and SCORE2/SCORE2-OP risk scores in terms of individual absolute risk scores. However, the SCORE2 risk system tends to categorize patients as higher risk compared to PREVENT system. In terms of predicting the likelihood of CAD based on CCTA findings, the PREVENT risk categories appear to be more accurate than those of the SCORE2 system. The SCORE2 system did not show a good association between its risk categories and CCTA findings, whereas the PREVENT system did. Our data are among the first in the literature to compare the SCORE2 and PREVENT risk assessment systems and to provide partial validation of the PREVENT score in assessing the likelihood of CAD.

## Figures and Tables

**Table 1:** Baseline demographic and laboratory data of all patients.

	All Patients (*n* = 171)
Age * (years)	53.7 ± 13.3
Women (*n*, %)	52 (30.4)
Body mass index ^†^ (kg/m^2^)	27 (25–30)
Systolic blood pressure * (mmHg)	132.2 ± 20.1
Diastolic blood pressure * (mmHg)	77.4 ± 11.9
Hypertension (*n*, %)	67 (39.2)
Diabetes mellitus (*n*, %)	37 (21.6)
Family history (*n*, %)	64 (37.4)
Smoking (*n*, %)	
Non-smoker	94 (55)
Smoker	64 (37.4)
Former smoker	13 (7.6)
Creatinine ^†^ (mg/dL)	0.86 (0.77–0.96)
eGFR ^†^ (mL/min/1.73 m^2^)	96 (86–105)
Hemoglobin ^†^ (gr/L)	15 (14–16)
Total cholesterol * (mg/dL)	231.7 ± 52.9
Triglyceride ^†^ (mg/dL)	150 (98–255)
LDL cholesterol * (mg/dL)	140.7 ± 43.4
HDL cholesterol ^†^ (mg/dL)	48 (39–59)

*: mean ± std. deviation; ^†^: median (interquartile range). **Abbreviations:** eGFR: estimated glomerular filtration rate; HDL: high-density lipoprotein; LDL: low-density lipoprotein.

**Table 2:** Computed tomography findings and risk categories according to the PREVENT and SCORE systems.

**Total Coronary Artery Calcium Score ^†^ (*n* = 171)**	0 (0–43)
**Grading of CAD According to Total Coronary Calcium Score (*n* = 171)**	
No evidence of CAD (0) (*n*, %)	87 (50.6)
Minimal (0–9) (*n*, %)	19 (11)
Mild (10–99) (*n*, %)	36 (20.9)
Moderate (100–399) (*n*, %)	17 (9.9)
Severe (400–999) (*n*, %)	3 (1.7)
Extensive (≥1000) (*n*, %)	9 (5.2)
**High-Grade Coronary Stenosis *(n, %)***	22 (12.9)
**PREVENT 10-Year ASCVD Risk Categories *(n, %)* (*n* = 171)**	
Low risk (<5%)	79 (46.2)
Borderline risk (5%–7.4%)	32 (18.7)
Intermediate risk (7.5%–19.9%)	51 (29.8)
High risk (≥20%)	9 (5.3)
**SCORE2 and SCORE2-OP CVD 10-Year Risk Categories *(n, %)* (*n* = 113)**	
Low to moderate risk	32 (28.3)
High risk	39 (34.5)
Very high risk	42 (37.2)

^†^: median (interquartile range). **Abbreviations:** CAD: coronary artery disease; CVD: cardiovascular disease; ASCVD: atherosclerotic cardiovascular disease; PREVENT: Predicting the risk of CVD EVENTS; SCORE2: Systemic Coronary Risk Estimation 2; SCORE2-OP: Systemic Coronary Risk Estimation 2-Older Persons.

**Table 3:** Patient classification according to the PREVENT and SCORE2 risk categories.

	SCORE2 and SCORE2-OP Risk Category		
PREVENT Risk Category	Low to Moderate Risk (*n* = 32)	High Risk (*n* = 39)	Very High Risk (*n* = 42)
**Low risk (*n*, %)**	32 (55.2)	22 (37.9)	4 (6.9)
**Borderline risk (*n*, %)**	0 (0)	15 (65.2)	8 (34.8)
**Intermediate risk (*n*, %)**	0 (0)	2 (6.5)	29 (93.5)
**High risk (*n*, %)**	0 (0)	0 (0)	1 (100)

Abbreviations: PREVENT: Predicting the risk of CVD EVENTS; SCORE2: Systemic Coronary Risk Estimation 2; SCORE2-OP: Systemic Coronary Risk Estimation 2-Older Persons.

**Table 4:** Differences in the computed tomographic findings according to PREVENT 10-year ASCVD risk score categories.

	Low Risk (*n* = 79)	Borderline Risk (*n* = 32)	Intermediate Risk (*n* = 51)	High Risk (*n* = 9)	*p*-Value
**Total CAC Score**					**<0.001**
Mean	18	15.8	270	955	
**Grading of CAD according to total CAC score**					**<0.001**
No evidence of CAD (*n*, %)	54 (68.4)	20 (62.5)	13 (25.5)	0 (0)	
Minimal (*n*, %)	7 (8.9)	2 (6.3)	8 (15.7)	2 (22.2)	
Mild (*n*, %)	13 (16.5)	8 (25)	15 (29.4)	0 (0)	
Moderate (*n*, %)	5 (6.2)	2 (6.3)	7 (13.7)	3 (33.3)	
Severe (*n*, %)	0 (0)	0 (0)	3 (5.9)	0 (0)	
Extensive (*n*, %)	0 (0)	0 (0)	5 (9.8)	4 (44.4)	
**High-grade coronary stenosis *(n,%)***	6 (7.6)	1 (3.1)	10 (19.6)	5 (55.6)	**<0.001**

**Abbreviations:** CAC: coronary artery calcium; CAD: coronary artery disease.

**Table 5:** Differences in computed tomography findings according to the SCORE2 and SCORE2-OP risk score categories.

	Low to Moderate Risk (*n* = 32)	High Risk (*n* = 39)	Very High Risk (*n* = 42)	*p*-Value
**Total CAC score**				0.3
Mean	40.3	15.8	145.7	
**Grading of CAD according to total CAC score**				0.051
No evidence of CAD (*n*, %)	20 (62.5)	19 (48.7)	15 (35.7)	
Minimal (*n*, %)	0 (0)	7 (17.9)	8 (19)	
Mild (*n*, %)	7 (21.9)	11 (28.2)	12 (28.6)	
Moderate (*n*, %)	5 (15.6)	2 (5.1)	3 (7.1)	
Severe (*n*, %)	0 (0)	0 (0)	2 (4.8)	
Extensive (*n*, %)	0 (0)	0 (0)	2 (4.8)	
**High-grade coronary stenosis *(n,%)***	4 (12.5)	2 (5.1)	6 (14.3)	0.37

**Abbreviations:** CAC: coronary artery calcium; CAD: coronary artery disease.

## Data Availability

Data available in a publicly accessible repository.

## References

[1] Visseren F.L.J., Mach F., Smulders Y.M., Carballo D., Koskinas K.C., Bäck M., Benetos A., Biffi A., Boavida J.M., Capodanno D. (2021). 2021 ESC Guidelines on cardiovascular disease prevention in clinical practice. Eur. Heart J..

[2] Arnett D.K., Blumenthal R.S., Albert M.A., Buroker A.B., Goldberger Z.D., Hahn E.J., Himmelfarb C.D., Khera A., Lloyd-Jones D., McEvoy J.W. (2019). 2019 ACC/AHA Guideline on the Primary Prevention of Cardiovascular Disease: A Report of the American College of Cardiology/American Heart Association Task Force on Clinical Practice Guidelines. Circulation.

[3] Manjunath G., Tighiouart H., Ibrahim H., MacLeod B., Salem D.N., Griffith J.L., Coresh J., Levey A.S., Sarnak M.J. (2003). Level of kidney function as a risk factor for atherosclerotic cardiovascular outcomes in the community. J. Am. Coll. Cardiol..

[4] Mongraw-Chaffin M.L., Peters S.A.E., Huxley R.R., Woodward M. (2015). The sex-specific association between BMI and coronary heart disease: A systematic review and meta-analysis of 95 cohorts with 1·2 million participants. Lancet Diabetes Endocrinol..

[5] Ndumele C.E., Neeland I.J., Tuttle K.R., Chow S.L., Mathew R.O., Khan S.S., Coresh J., Baker-Smith C.M., Carnethon M.R., Després J.P. (2023). American Heart Association. A Synopsis of the Evidence for the Science and Clinical Management of Cardiovascular-Kidney-Metabolic (CKM) Syndrome: A Scientific Statement from the American Heart Association. Circulation.

[6] Nelson A.J., Pagidipati N.J., Aroda V.R., Cavender M.A., Green J.B., Lopes R.D., Al-Khalidi H., Gaynor T., Kaltenbach L.A., Kirk J.K. (2021). Incorporating SGLT2i and GLP-1RA for Cardiovascular and Kidney Disease Risk Reduction: Call for Action to the Cardiology Community. Circulation.

[7] Khan S.S., Matsushita K., Sang Y., Ballew S.H., Grams M.E., Surapaneni A., Blaha M.J., Carson A.P., Chang A.R., Ciemins E. (2024). Chronic Kidney Disease Prognosis Consortium and the American Heart Association Cardiovascular-Kidney-Metabolic Science Advisory Group. Development and Validation of the American Heart Association’s PREVENT Equations. Circulation.

[8] https://professional.heart.org/en/guidelines-and-statements/prevent-calculator.

[9] Perrone-Filardi P., Achenbach S., Möhlenkamp S., Reiner Z., Sambuceti G., Schuijf J.D., Van der Wall E., Kaufmann P.A., Knuuti J., Schroeder S. (2011). Cardiac computed tomography and myocardial perfusion scintigraphy for risk stratification in asymptomatic individuals without known cardiovascular disease: A position statement of the Working Group on Nuclear Cardiology and Cardiac CT of the European Society of Cardiology. Eur. Heart J..

[10] Wormser D., Kaptoge S., Di Angelantonio E., Wood A.M., Pennells L., Thompson A., Sarwar N., Kizer J.R., Lawlor D.A., Emerging Risk Factors Collaboration (2011). Separate and combined associations of body-mass index and abdominal adiposity with cardiovascular disease: Collaborative analysis of 58 prospective studies. Lancet.

[11] SCORE2 working group and ESC Cardiovascular risk collaboration (2021). SCORE2 risk prediction algorithms: New models to estimate 10-year risk of cardiovascular disease in Europe. Eur. Heart J..

[12] Sud M., Sivaswamy A., Austin P.C., Abdel-Qadir H., Anderson T.J., Khera R., Naimark D.M.J., Lee D.S., Roifman I., Thanassoulis G. (2024). Validation of the European SCORE2 models in a Canadian primary care cohort. Eur. J. Prev. Cardiol..

[13] Choi J., Sung S., Park S.K., Park S., Kim H., Cho M.C., Williams B., Lee H.Y. (2023). SCORE and SCORE2 in East Asian Population: A Performance Comparison. JACC Asia.

[14] Svinin G.E., Kutsenko V.A., Shalnova S.A., Yarovaya E.B., Imaeva A.E., Balanova Y.A., Kapustina A.V., Muromtseva G.A., Drapkina O.M. (2024). Validation of SCORE2 on a sample from the Russian population and adaptation for the very high cardiovascular disease risk region. PLoS ONE.

[15] Lin J.S., Evans C.V., Johnson E., Redmond N., Coppola E.L., Smith N. (2018). Nontraditional Risk Factors in Cardiovascular Disease Risk Assessment: Updated Evidence Report and Systematic Review for the US Preventive Services Task Force. JAMA.

